# Supplementary material for: Complexome profiling of the Chlamydomonas psb28 mutant reveals TEF5 as an early PSII assembly factor
Source: Plant Cell. 2025 Mar 17;37(6):koaf055. doi: 10.1093/plcell/koaf055 (PMC12164590; doi:10.1093/plcell/koaf055)
Supplement: koaf055_Supplementary_Data [file koaf055_supplementary_data.zip › psb28_interactive.zip!/plots/intensity/qualityPlots/peptides/TEF4_Cre02.g111450.t1.2.html]

### Cre02.g111450.t1.2

Trivial name: TEF4  
SeqCoverage [median]: 27.50  
Mass: 26.62 kDa  
  
 

| Sequence | MissedCleavage | Mass [Da] | wtIntensity | muIntensity | Score |
| --- | --- | --- | --- | --- | --- |
| FNEQIK | 0 | 777.40 | 5717.05 | 1804.07 | 139.92 |
| GYEMRPEDKPVEAASK | 2 | 1805.86 | 3998.10 | 0.00 | 61.73 |
| SLQAAYR | 0 | 807.42 | 4567.98 | 535.38 | 94.41 |
| TPMAAAEPTLQR | 0 | 1284.65 | 4171.85 | 2036.20 | 119.16 |
| VLTEGLAPSVAHLDR | 0 | 1576.86 | 11705.50 | 6174.27 | 85.46 |
| VQSVSPEEAAR | 0 | 1171.58 | 8927.91 | 2989.60 | 242.92 |
|  |  |  | 39088.4 | 13539.5 |  |
